# Assessment of left and right ventricular volumes and function with treadmill exercise stress cardiovascular magnetic resonance

**DOI:** 10.1186/1532-429X-13-S1-P29

**Published:** 2011-02-02

**Authors:** Leah Geyer, Subha V Raman, Vijay Balasubramanian, Beth McCarfthy, Orlando P Simonetti, Stephen C Cook

**Affiliations:** 1 The Ohio State University, Columbus, OH, USA

## Introduction

Pharmacologic stress testing with cardiovascular magnetic resonance (CMR) is employed to assess cardiac function and perfusion in various conditions. However, the response elicited may not compare to that obtained during physical exercise. Advances in CMR technology now include a MR-compatible treadmill proven feasible in evaluation of ischemic heart disease.

## Purpose

The aims of this study were: (1) compare biventricular volumes and function obtained with gated and real-time imaging techniques at rest and (2) biventricular volumes and function measured after peak treadmill exercise stress CMR in healthy volunteers.

## Methods

Ten young adults (mean age 26.1 ± 8.4 years); seven men, were prospectively enrolled. All examinations were performed with a 1.5T scanner (Siemens Avanto) and 32-channel phased array coil (Rapid MRI, Columbus). Ventricular volumes were obtained at rest with gated (SSFP) and real-time imaging (TSENSE). Nine to 10 contiguous short axis and axial slices provided coverage of left and right ventricular volumes respectively. Endocardial borders were manually segmented and ejection fraction (EF) calculated as the difference between end-diastolic volume (EDV) and end-systolic volume (ESV) divided by EDV. Segmented breath-hold Cine SSFP imaging was performed at rest (TE 1.0ms; TR 3ms; temporal resolution 39ms; flip angle (FA) 69°, bandwith (BW) 930Hz/pixel and slice thickness 8mm). Non-triggered real-time TSENSE image acquisition was performed at rest and stress (TE 1.0msec; TR 2.3msec; temporal resolution 46ms, FA 62°, BW 1360Hz/pixel and slice thickness 8mm). Patients underwent treadmill exercise CMR with 12-lead ECG system (Cardiosoft, GE) to achieve a goal of > 85% age-predicted maximum heart rate (APMHR). Real-time imaging was performed immediately upon cessation of exercise.

## Results

All 10 young adults successfully completed the treadmill exercise CMR examination, achieving a maximal heart rate 186.9 ± 11.1bpm (96.5 ± 5.7% APMHR). From the end of exercise to the start of imaging, on average 20.5± 4.1s elapsed. Results of left and right ventricular volumes and function obtained with SSFP and TSENSE techniques at rest and stress are listed in Table 1. ECG data revealed no arrhythmias or ST segment changes during exercise.

**Table 1:** Ventricular Volumes and Function Obtained at Rest and Stress Measured by Segmented and Real-Time Seq

	End Diastolic Volume (mL)	End Systolic Volume (mL)	Ejection Fraction (%)
SSFP Rest RV	117.4	56.3	52
TSENSE Rest RV	133.1	55.5	58.4
TSENSE Stress RV	99.2	30.9	68.7
SSFP Rest LV	136.3	57.6	57.6
TSENSE Rest LV	126.0	52.6	58.2
TSENSE Stress LV	125.3	30.1	76

## Conclusions

In-room treadmill exercise stress CMR provides a novel and feasible technique to assess right and left ventricular function. This study not only demonstrates the equivalence of real-time and segmented techniques, but also provides the framework to measure function at peak exercise stress. Importantly, this study describes the biventricular response to treadmill exercise in healthy volunteers.

